# Recent Progress of Iron-Based Magnetic Absorbers and Its Applications in Elastomers: A Review

**DOI:** 10.3390/ma17164058

**Published:** 2024-08-15

**Authors:** Wanting Xu, Na Liu, Zhongchen Lu

**Affiliations:** 1 School of Mechanical and Automotive Engineering, South China University of Technology, Guangzhou 510640, China; 202221004154@mail.scut.edu.cn; 2 Key Laboratory of Advanced Energy Storage Materials of Guangdong Province, Guangzhou 510640, China; 202310186875@mail.scut.edu.cn; 3 School of Materials Science and Engineering, South China University of Technology, Guangzhou 510640, China

**Keywords:** microwave-absorbing materials, ferromagnetic absorbers, carbonyl iron powder, electromagnetic properties, components

## Abstract

As a result of continuing scientific and technological progress, electromagnetic waves have become increasingly pervasive across a variety of domains, particularly within the microwave frequency range. These waves have found extensive applications in wireless communications, high-frequency electronic circuits, and several related fields. As a result, absorptive materials have become indispensable for dual-use applications across both the military and civilian domains because of their exceptional electromagnetic wave absorption properties. This paper, beginning with the operating mechanisms of absorptive materials, aims to provide an overview of the strategies that have been used to enhance the absorption performance of iron-based magnetic absorbers (IBMAs) and discuss the current research status of absorptive material components. The fabrication of a ferromagnetic absorber in terms of morphology, heterointerface coupling, and macrostructural enhancements and the effect of powder characteristics on their electromagnetic properties are discussed. Additionally, the application of IBMAs in elastomers is summarized. Finally, this paper summarizes the limitations of existing ferromagnetic absorber materials and offers a perspective on their potential future developments. The objective of the ongoing research is to fabricate absorptive components that have thin profiles, lightweight construction, wide absorption frequency ranges, and strong absorption capabilities.

## 1. Introduction

The microwave frequency spectrum encompasses electromagnetic waves with frequencies in the range of 300 MHz to 3000 GHz and corresponding wavelengths that span from 1 m to 0.1 mm. Microwaves have unique properties that are unlike those of waves in other frequency bands, including quasi-optical properties and enhanced penetration capabilities. To some extent, the control and application of microwave fields can provide indications of a country’s national defense and military capabilities and of its proficiency in information and communication technology [1,2,3]. The generation, transmission, reception, and control of microwaves are of major significance for the national defense and civil fields [4,5,6,7,8]. Microwave absorptive materials are functional materials that are designed to convert electromagnetic energy into other forms of energy through electromagnetic loss processes, thereby achieving attenuation of incident electromagnetic waves. Although they were originally developed to provide radar stealth properties in military applications, these materials have also been used in microwave electronic communications and other fields in recent years to reduce or prevent electromagnetic interference within electronic equipment and to protect individuals from the potential health risks of electromagnetic radiation [9].

Absorptive materials are typically created by combining an absorber with a matrix material. The absorber is the essential component and is responsible for electromagnetic absorption, while the matrix material determines the absorptive material’s structure, its load-bearing capacity, and its adaptability to various environmental conditions. As a result of their high saturation magnetization (*M_s_* = 218 emu/g), Curie temperature, Snoek limit, and electromagnetic loss characteristics, IBMAs have demonstrated considerable potential to address various electromagnetic challenges [10,11,12,13]. Moreover, the raw materials for these substances are plentiful and inexpensive, which facilitates mass production and widespread use. Furthermore, the magnetic properties of iron-based absorptive materials can be modulated by an external magnetic field, offering the potential for the dynamic adjustment of their absorptive capabilities [14,15,16]. Presently, research on iron-based absorptive materials is primarily focused on enhancing their efficiency of absorption, broadening their bandwidth, and reducing their weight. Researchers are continually refining the absorptive properties by varying the materials’ composition, microstructure, morphology, and the methods of compositing [13,17,18,19,20]. For example, Hu and et al. designed and prepared one- and two-dimensional Fe_0.9_Co_0.1_ nanoalloys with high permeability through an organic templating method combined with a constrained transformation strategy. It can achieve a bandwidth of 4.2 GHz at a thickness of 0.7 mm [21]. Additionally, in practical applications, IBMAs are often used as coating fillers [22,23]. Despite their widespread use in electromagnetic wave absorption, they exhibit shortcomings such as poor broadband performance, heavy weight, and temperature sensitivity. In contrast, elastomer-based composite absorbers, which can be fashioned into films or foams, facilitate lightweight design and possess excellent mechanical flexibility and structural tunability, effectively compensating for the deficiencies of absorptive coatings [24,25]. These materials are typically created by combining magnetic or conductive fillers with an elastic base, such as polymers, aiming to achieve a balance between efficient wave absorption and mechanical flexibility. Such a design caters to the need for materials that are lightweight, thin, flexible, and capable of wide-bandwidth absorption, which is particularly relevant given the rapid advancement of portable electronic devices and flexible wearable technology [26]. The strength of elastomeric absorptive materials lies in their ability to maintain electromagnetic wave absorption efficiency while conforming to surfaces with complex shapes and dynamic deformations, thus providing greater design flexibility and expanding application possibilities. Moreover, these materials are generally characterized by their low density and cost-effectiveness, ease of processing and manufacturing, and the capability to fine-tune their absorptive properties by adjusting the composite’s composition and structure. This allows them to meet diverse application needs across various frequency ranges and environmental conditions [27,28,29]. Consequently, research on elastomeric absorptive materials extends beyond their absorption capabilities to include a thorough examination of their mechanical strength, environmental resilience, and manufacturing techniques, all aimed at creating more effective and practical solutions for electromagnetic interference [30,31,32]. For example, Prakash’s study revealed that adding 20 wt% iron–nickel–nanographite nanopowder to silicone rubber not only increased its tensile strength by about 60% but also enabled wide-frequency absorption in the 8–18 GHz range. Furthermore, even after 16 h of artificial UV-induced weathering, the nanopowder-enhanced composite rubber showed negligible damage compared with the pure silicone rubber [33]. This paper summarizes the design and synthesis strategies employed for IBMAs and reviews their application within elastomer matrices. Furthermore, gaps in existing research are also identified, and a forward look to potential future developments in the field is provided.

## 2. The Principles of Electromagnetic Wave Absorption

**Figure 1:**
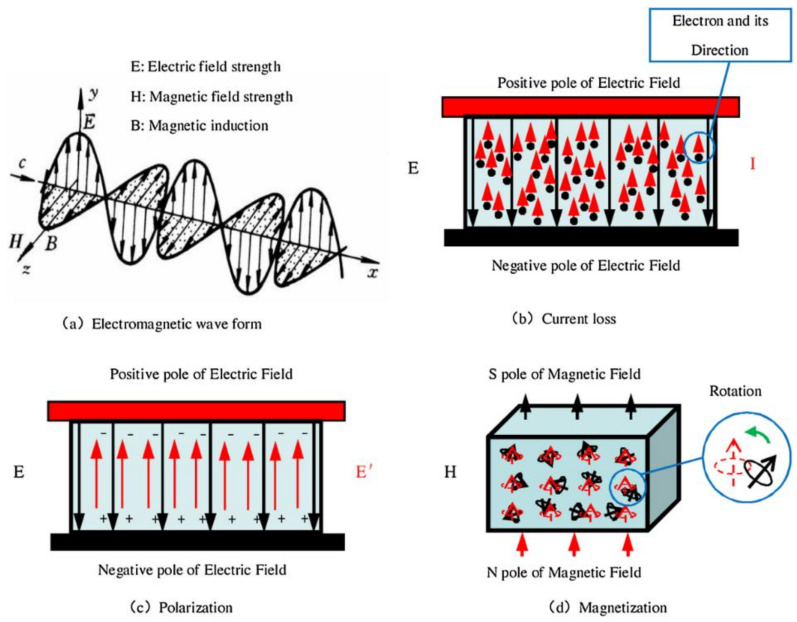
Diagrams illustrating the principles of electromagnetic wave absorption [35].

As illustrated in Figure 1a, microwaves are dynamic electromagnetic waves composed of alternating electric fields and alternating magnetic fields [34,35]. When the dynamic electromagnetic fields of microwaves are applied to absorptive materials, the materials experience current/resistive losses (Figure 1b) (due to the abundance of free electrons within conductive materials, they accelerate under the influence of an electric field and interact with the material’s lattice structure, converting into thermal energy); polarization/dielectric losses (Figure 1c) (under the external electric field, the positive and negative charge centers within the dielectric material undergo relative displacement, resulting in a macroscopic electric dipole moment); and magnetic losses (Figure 1d) (when magnetic materials are exposed to a changing magnetic field, the internal magnetic domains reorient to respond to the changes in the external magnetic field, resulting in energy loss). These processes lead to the conversion of the electromagnetic energy into heat and ultimately result in the absorption of the microwaves [36]. The quality of an absorptive material is determined by its capacity to absorb and dissipate microwaves efficiently. Exceptional absorptive materials should be able to maximize the absorption and dissipation of microwaves through a variety of mechanisms.

Researchers have developed a variety of methods and materials to meet the demands of multiple application scenarios. Commonly used absorptive materials are listed in Table 1 below.

**Table 1:** Types of absorptive materials.

Electromagnetic Wave Absorbing Material		Features
Carbon-based material	Hollow carbon microspheres [37], carbon nanotubes [38,39], layered carbon [40], porous carbon [41], reduced graphene oxide [42], carbon-based composites [43,44,45]	Lightweight, high conductivity, large specific surface area
Conductive polymer-based materials	Polyaniline matrix composites [46,47], polypyrrole matrix composites [19,48], polythiophenyl matrix composites [49]	Low density, High mechanical performance, controllable electrical conductivity [50], excellent corrosion resistance
Iron-based material	Carbonyl iron [51], ferrites [52], soft magnetic alloys [53]	Excellent magnetic properties [54], high Snoek limit, high Curie temperature, strong magnetic loss capability
Ceramic base material	SiC [55,56], Si_3_N_4_ [57], BaTiO_3_ [58]	High hardness, high-temperature resistance, corrosion resistance, low density [59]
New generation of absorber	MOF [60,61,62], MXene [63,64], metamaterial [65,66]	Strong designability [65]

**Figure 2:**
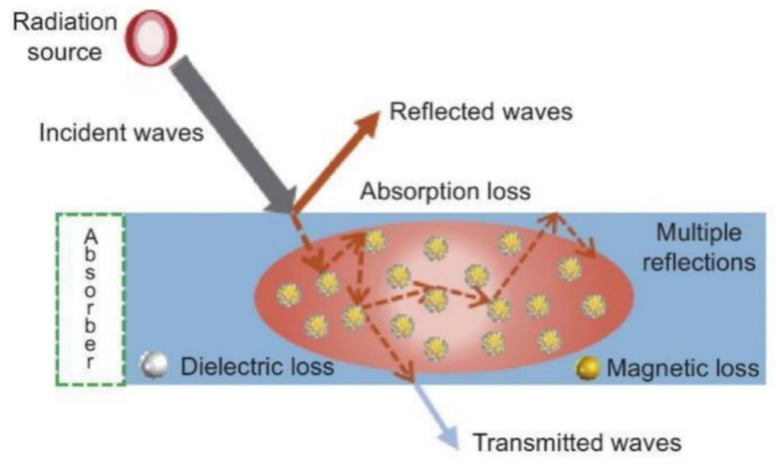
Propagation path of electromagnetic waves [68].

When electromagnetic waves strike the surface of an absorptive material, some of the incident waves are reflected, some actually enter the material and are absorbed, and the remainder simply pass through. The entire electromagnetic wave propagation path during this process is shown schematically in Figure 2. The absorption and attenuation behavior of electromagnetic waves in absorptive materials is primarily influenced by two factors. First, the penetration of electromagnetic waves into an absorptive material is determined by that material’s impedance matching characteristics. Second, the ability of the electromagnetic energy to be converted rapidly into thermal or other forms of energy within the material after entering the material is dependent on the material’s microwave loss properties.

From a microscopic perspective, when a material is exposed to an electromagnetic field, the charged particles in the material undergo changes in their distribution because of the influence of the electromagnetic field. These changes are observed at the macroscopic level as the material’s response to the electromagnetic field and encompass polarization, magnetization, and conduction effects [67]. These responses can be quantified using parameters such as dielectric constant (*ε*), magnetic permeability (*μ*) and electrical conductivity (*σ*).

### 2.1. Impedance Matching and Reflection Loss (RL)

According to electromagnetic wave theory, when an electromagnetic wave moves from one medium to another, if the wave impedances in the two media are exactly matched, the wave will then pass through the interface seamlessly without the occurrence of any reflection. To minimize reflection and improve the absorption ability of these materials, it is necessary to investigate the impedance matching conditions for each material. Using transmission line theory [69], the input impedance at the interface of an absorber material can be calculated using the complex permittivity and the complex permeability at a known material thickness, as follows:

(1) $$Z_{in}=Z_{0}\sqrt{\frac{\mu_{r}}{\varepsilon_{r}}}tanh(j\frac{2\pi f}{c}d\sqrt{\mu_{r}\varepsilon_{r}})$$

(2) $$\varepsilon_{r}=\frac{1}{\varepsilon_{0}}\left(\varepsilon^{\prime }-j\varepsilon^{\prime \prime }\right)=\frac{\varepsilon^{\prime }}{\varepsilon_{0}}(1-jtan\delta_{\varepsilon })$$

(3) $$\mu_{r}=\frac{1}{\mu_{0}}\left(\mu^{\prime }-j\mu^{\prime \prime }\right)=\frac{\mu^{\prime }}{\mu_{0}}(1-jtan\delta_{\mu })$$

In the equations given above,$\;Z_{0}$ is the inherent impedance of free space, where $Z_{0}$ = 377 Ω, $Z_{in}$ represents the impedance of the absorptive material, f is the microwave frequency, c signifiesthe speed of light,andd is the absorptive material thickness. The equations illustrate that the microwave attenuation capacity is predominantly influenced by the complex permittivity ($\varepsilon_{r}=\varepsilon^{\prime }-j\varepsilon^{\prime \prime }$). The equations also illustrate that the microwave attenuation capacity is predominantly influenced by the complex permeability ($\mu_{r}=\mu^{\prime }-j\mu^{\prime \prime }$).

**Figure 3:**
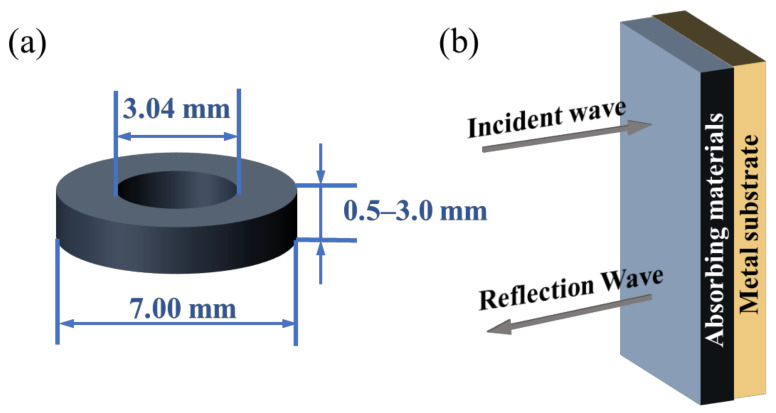
(**a**) Coaxial samples used to measure the electromagnetic parameters. (**b**) Single-layer microwave absorber model.

The real part of the permittivity $\varepsilon^{\prime }$ represents the material’s polarization response to the alternating electric fields and its ability to store electric charge, while the imaginary part $\varepsilon^{\prime \prime }$ represents the material’s ability to absorb electromagnetic waves due to losses. Similarly, the real part of the permeability $\mu^{\prime }$ represents the material’s magnetization response to alternating magnetic fields, while the imaginary part $\mu^{\prime \prime }$ demonstrates the magnetic loss ability of the material. Furthermore, as per Equations (2) and (3), the realization of nonreflective absorption obviously necessitates the condition that the tangent of the dielectric loss angle is equal to the tangent of the magnetic loss angle. The measurement of the electromagnetic parameters is conventionally conducted by making coaxial ring samples from the absorptive materials and then using a vector network analyzer to obtain the measured values, as illustrated in Figure 3a.

Reflection loss (*RL*) is an important indicator for the evaluation of the performance of absorptive materials. In a single-layer model (as illustrated in Figure 3b), the *RL* of an absorptive material can be expressed as follows:

(4) $$RL=20lg|\frac{Z_{in}-Z_{0}}{Z_{in}+Z_{0}}|$$

Smaller *RL* values correspond to higher absorber efficiency.

### 2.2. Electromagnetic Wave Loss Mechanism

Based on their distinct electromagnetic wave loss mechanisms, absorptive materials can be classified as dielectric loss materials, magnetic loss materials, or composite loss materials.

#### 2.2.1. Dielectric Loss

**Figure 4:**
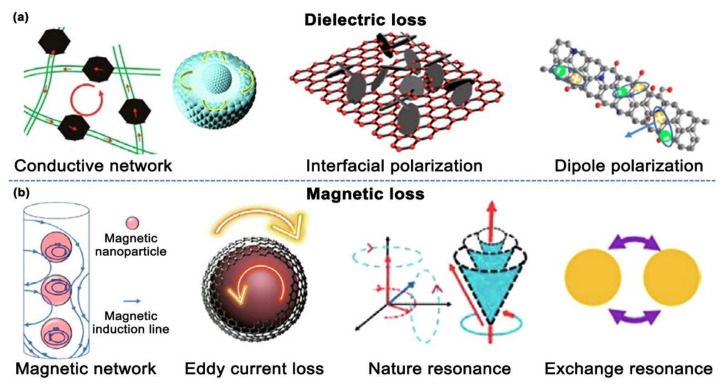
Illustration of (**a**) dielectric and (**b**) magnetic losses in absorptive materials [76].

Within the GHz range, the main factors contributing to the dielectric losses are the conduction loss, dipole polarization, and interface polarization [70], as illustrated in Figure 4a.

Conduction losses occur as electromagnetic waves pass through the absorptive materials, where the wave energy is transformed into an electrical current. During current flow, the resistance within the material generates Joule heating, which leads to the dissipation of the electromagnetic wave energy. Materials with high electrical conductivity, such as conductive polymers (e.g., polypyrrole, polyaniline) and carbon-based materials (e.g., carbon fibers, graphene, carbon nanotubes), exhibit conduction losses. The conduction loss in these materials can be described based on the principles of free electron theory [71], as follows:

(5) $$\varepsilon_{c}^{\prime \prime }=\frac{\sigma }{2\pi f\varepsilon_{0}}$$

In this equation, σ is the electrical conductivity, f is the frequency of the electromagnetic wave, and $\varepsilon_{0}$ is the permittivity of free space. The equation clearly demonstrates that the conduction loss increases with increasing electrical conductivity.

The polarization loss encompasses both dipole polarization and interface polarization [72]. Dipole polarization is caused by the presence of dipoles composed of positive and negative charges within the material. When such a material is exposed to an alternating electromagnetic field, these internal positive and negative charges move toward opposite ends of the material, thus creating the electric dipoles under the influence of the electromagnetic field. Under high-frequency electromagnetic field conditions, the reorientation of the dipoles lags behind the changes in the applied electromagnetic field; this phenomenon is known as polarization relaxation. This relaxation process induces an electric field within the material, which leads to interactions with the alternating electromagnetic field and causes energy losses in the electromagnetic waves. Interface polarization, in contrast, arises from differences between the compositions of two materials. Under the influence of an electromagnetic field, the uneven distribution of the charges on the two sides of a heterogeneous interface leads to the formation of a space charge layer that causes interface polarization [73].

According to the Debye equation, the real and imaginary components of a material’s dielectric constant can be expressed as [74] follows:

(6) $$\varepsilon^{\prime }=\varepsilon_{\infty }+\frac{\varepsilon_{s}-\varepsilon_{\infty }}{1+{\left(2\pi f\right)}^{2}\tau^{2}}$$

(7) $$\varepsilon^{\prime \prime }=\varepsilon_{c}^{\prime \prime }+\varepsilon_{p}^{\prime \prime }=\frac{\sigma }{2\pi f\varepsilon_{0}}+\frac{2\pi f(\varepsilon_{s}-\varepsilon_{\infty })}{1+4\pi^{2}f^{2}\tau^{2}}$$

In these equations, $\varepsilon_{s}$ stands for the fixed dielectric constant, $\varepsilon_{\infty }$ represents the dielectric constant at an infinite frequency, τ is the polarization relaxation time, and $\varepsilon_{p}^{\prime \prime }$ accounts for the polarization loss. When the electromagnetic field frequency matches the condition that $f=\frac{1}{2\pi \tau }$, the material is then subjected to polarization effects, which lead to a rapid reduction in $\varepsilon^{\prime }$ and the peaking of $\varepsilon^{\prime \prime }$. By combining and simplifying Equations (6) and (7), we then obtain the following expression:

(8) $${\left(\varepsilon^{\prime }-\frac{\varepsilon_{s}+\varepsilon_{\infty }}{2}\right)}^{2}+{\left(\varepsilon^{\prime \prime }\right)}^{2}={\left(\frac{\varepsilon_{s}-\varepsilon_{\infty }}{2}\right)}^{2}$$

By using $\varepsilon^{\prime }$ as the horizontal axis and $\varepsilon^{\prime \prime }$ as the vertical axis, a Cole–Cole semicircle diagram is created to illustrate the degree of relaxation polarization within a material. Each Cole–Cole semicircle corresponds to one or more of the Debye relaxation processes [75].

#### 2.2.2. Magnetic Loss

When compared with dielectric losses, magnetic losses involve a wider range of mechanisms, including eddy current losses, hysteresis losses, domain wall resonance losses, and ferromagnetic resonance losses that are induced by natural/exchange resonance [77], as depicted in Figure 4b. The specific magnetic loss mechanism is dependent on the frequency range of the alternating magnetic field and the material’s various coupling modes.

When magnetic absorbing materials are subjected to an alternating magnetic field, currents are generated within the conductor through electromagnetic induction. The resulting thermal effects dissipate the energy from the alternating magnetic field. These eddy current losses can be determined using the following equation [78]:

(9) $$C_{0}=\frac{2}{3}\pi \mu_{0}d^{2}\sigma$$

In this equation, $\mu_{0}$ represents the vacuum permeability and d is the material thickness. From this equation, we can deduce that if the material thickness remains constant, then the eddy current losses do not vary with changes in the magnetic field frequency. However, excessive eddy current losses can cause the magnetic field to be concentrated at the material’s surface, thus making it difficult for the electromagnetic waves to penetrate the material’s interior. In general, eddy current losses do not exist in isolation in magnetic absorptive materials. Within the range of 2–18 GHz, magnetic materials also exhibit ferromagnetic resonance. When a ferromagnetic material is exposed to an alternating magnetic field, a dynamic magnetization process occurs [79]. Subsequently, some of the material’s domain walls undergo irreversible rotation or displacement. During this phase, the field induced within the material lags behind the external magnetic field, thus creating a phase difference. The resulting energy is converted into heat and dissipated, and this process is known as hysteresis. The strength of the hysteresis loss is determined by the size of the hysteresis loop area; in other words, a larger hysteresis loop area corresponds to more significant hysteresis losses.

Ferromagnetic resonance describes the strong resonance absorption that occurs in ferromagnetic materials when they are exposed to both a constant magnetic field and an alternating magnetic field. This resonance causes a peak value to occur for the complex magnetic susceptibility or magnetic permeability. Natural resonance, in contrast, is a specific form of the ferromagnetic resonance. In certain ferromagnetic materials, including single crystals and polycrystalline materials, a resonance phenomenon that is similar to ferromagnetic resonance can occur even in the absence of a constant magnetic field. This phenomenon is known as natural resonance, and it typically occurs at low frequencies [80].

### 2.3. Snoek Limit

The microwave magnetic permeability of a material is intimately connected to the phenomenon of precession of the magnetic dipoles within the material in response to the application of an external electromagnetic field. In practical scenarios involving electromagnetic waves, when the precession frequency of the magnetic dipoles aligns with the external magnetic field frequency, the dominant mechanism for the magnetic losses is natural resonance. This specific frequency is known as the cutoff frequency and corresponds to the point at which the imaginary component of the magnetic permeability reaches its maximum value. This phenomenon is influenced by the material itself and is manifested in the initial magnetic permeability, which is denoted by $\mu_{i}$ (the real component of the magnetic permeability at a zero magnetic field frequency). The resonance frequency is inversely proportional to $\mu_{i}$, and this correlation is known as the Snoek limit relationship [81]. For spherical magnetic metal micro-powder particles, this relationship can be expressed using the following equation:

(10) $$(\mu_{i}-1)f_{r}=\frac{1}{3\pi }\gamma M_{s}$$

In this equation, $\mu_{i}$ is the initial magnetic permeability, $f_{r}$ is the natural resonance frequency, γ is the gyromagnetic ratio, and $M_{s}$ is the saturation magnetization.

From Equation (10), as the frequency of the applied magnetic field increases, the magnetic permeability values of magnetic metal materials will decrease continuously and will continue to be constrained by the Snoek limit. To achieve broad and effective electromagnetic wave absorption across a wide frequency range, it is thus essential to explore methods that can surpass the Snoek limit and allow the magnetic permeability to remain consistently high across the entire electromagnetic wave spectrum.

## 3. Magnetic Loss-Type Iron-Based Absorbers

Magnetic loss-type iron-based absorbers mainly include ferrite materials, iron-based metal powders, and their alloys. These materials exhibit strong electromagnetic wave absorption capabilities because of their superior magnetic properties, including higher Snoek cutoff frequencies, saturation magnetization values, and Curie temperatures. However, these iron-based magnetic particles also have the disadvantages of easy oxidation, poor impedance matching, high density, and obvious skin effects [82]. To achieve outstanding material absorption performances, it is essential to manipulate the electromagnetic properties of these materials to meet the demands of their various applications in different situations. The electromagnetic characteristics of iron-based metal powders depend on numerous factors, including their chemical compositions, grain sizes, internal stresses, particle shapes, grain orientations, and other related aspects. In current research, two common strategies are being used to improve the absorption capabilities of these powders: designing their structural morphologies and creating composite materials with multiple components. In this paper, the current research is divided into two parts: morphology design and multi-interface coupling.

### 3.1. Morphology Design of Ferromagnetic Particles and Their Absorption Performances

The natural resonance frequency and the relative magnetic permeability of isotropic magnetic particles with spherical morphologies follow the Snoek limit formula. Ferromagnetic materials with nonspherical shapes exhibit varying degrees of ease of magnetization along different directions. This phenomenon is referred to as the shape anisotropy of ferromagnetic materials. As the shape anisotropy increases, the demagnetization factor along the corresponding direction decreases, which makes the particles increasingly easy to magnetize, and a higher magnetic permeability is thus obtained. However, when the particle shape changes, the demagnetization factor will also change, and this causes the particles to exceed the Snoek limit. Equation (10) can then be modified as follows [81]:

(11) $$\left(\mu^{,}-1\right)f_{r}^{2}={\left(2.8\right)}^{2}4\pi \left[H_{k}+4\pi M_{s}(D_{\tau }-D_{e})\right]$$

In the modified equation, $H_{k}$ represents the intrinsic anisotropy field; $D_{\tau }\;and{\;D}_{e}$ are the demagnetization factors (for sheet-like particles, $D_{\tau }=1,\;D_{e}=0$; for spherical particles, $D_{\tau }=D_{e}=1/3$; and for fiber-like particles, $D_{\tau }=1/2,D_{e}=0$). Equation (11) indicates that both sheet-like and fiber-like particles can achieve higher magnetic permeabilities when compared with spherical particles. Researchers have created various forms of magnetic metal powders in an innovative manner by moving beyond the traditional spherical shape.

#### 3.1.1. Porous Spherical Absorbers

**Figure 5:**
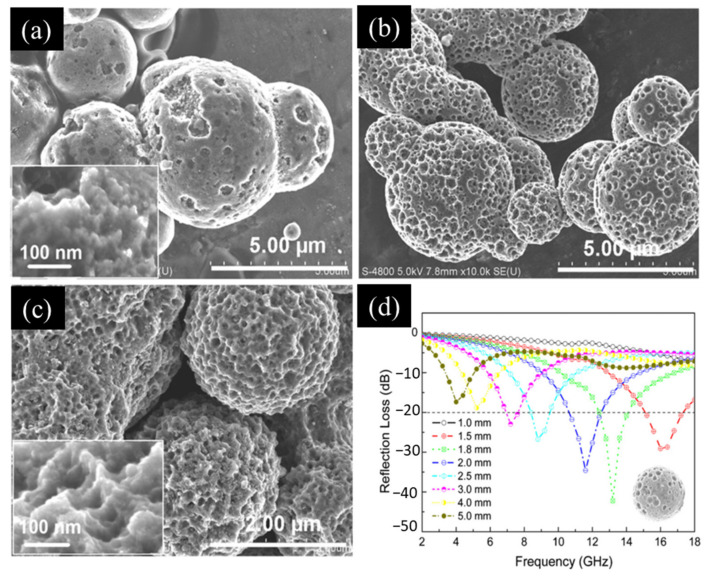
(**a**) Scanning electron microscope (SEM) image of coating with a 15 min etching time. (**b**) SEM image of coating with a 30 min etching time. (**c**) SEM image of a coating with a 60 min etching time. (**d**) Calculated *RL* versus frequency characteristics of wax composite coatings containing 20 vol.% of PIPs with various thicknesses. The plots were obtained with an etching time *t* of 15 min [83].

Porous microspheres have attracted significant research interest in recent years because of their low density and high surface area. Tong and colleagues applied a straightforward corrosion method that used citric acid and a ferric chloride solution to fabricate porous carbonyl iron particles (PIPs). The morphology of these PIPs was controlled by adjusting the Fe^3+^ concentration and the corrosion time, as illustrated in Figure 5a–c. Their findings indicated that a 15 min corrosion time outperformed a 60 min time in terms of the resulting microwave absorption. In wax-based materials with a 20% PIP content, when the coating thickness ranged from 1.5 to 3 mm, the *RL* remained at or below −20 dB across the frequency range from 7.2 to 17.2 G Hz. Notably, at 13.2 GHz, the *RL* reached a minimum of −42.2 dB, as depicted in Figure 5d. This exceptional microwave absorption performance from the PIPs can be attributed to the enhanced polarization loss and the high specific surface area of the materials, along with the involvement of various absorption mechanisms, including electromagnetic wave interference, which occurred within the pores [83].

#### 3.1.2. Lamellar Absorbers

The natural morphology of ferromagnetic particles is typically spherical. However, lamellar (sheet-like) particles are preferred to spherical particles for absorbers because they offer a reduced skin effect and higher complex magnetic permeability [18]. To produce lamellar iron particles, researchers commonly use ball milling techniques, and the creation of lamellar flake-shaped carbonyl iron (FCI) particles is the most straightforward and widely used approach. During the ball milling process, the powder goes through two distinct process stages: micro-forging and cold welding. The mechanical stress applied by the milling balls causes the powder particles to flatten, resulting in a rapid reduction in the particle thickness and a gradual increase in the lamellar diameter. Previous research has established that the morphology and the properties of lamellar carbonyl iron are influenced by several factors. These factors include the ball milling process duration, the ratio of balls to powder, the initial particle size, and the annealing temperature used [84].

A research conducted by Wen and his team showed that lamellar carbonyl iron powder has a higher dielectric constant when compared with that of spherical carbonyl iron powder. There are several reasons why this phenomenon occurs: (1) anisotropy (lamellar carbonyl iron particles demonstrate anisotropic behavior, which contributes to their ability to surpass the Snoek limit and enables enhanced microwave absorption properties); (2) reduced skin depth (thinner lamellae have smaller skin depths, which effectively limit the depth to which eddy current losses can occur, and this reduction in their eddy current losses contributes to their improved microwave absorption characteristics); and (3) alterations in magnetic moment orientation (changes in the particle shape cause variations in the orientations of the magnetic moments within the material). Additionally, the spatial charge polarization is also influenced by the particle shape alterations. A combination of these effects results in a shift in the frequency at which the material exhibits its minimum *RL* toward lower frequencies, thus further enhancing its performance in terms of microwave absorption applications [85]. On the microscale, the manipulation of the grain size and the internal strain in lamellar carbonyl iron plays a critical role in enhancing the material’s remarkable microwave absorption properties. During the ball milling process, the powder particles experience both fragmentation and mechanical forging, thus leading to a reduction in their grain size. However, this process introduces internal strain to the material simultaneously. Subsequently, the heat treatment process causes an increase in grain size while also effectively eliminating this introduced strain. These microstructural modifications contribute significantly to the improvement in the material’s microwave absorption performance. Wang and his team developed a novel approach that combined the benefits of two existing processing techniques to enhance the microwave absorption properties of lamellar carbonyl iron powders. Their research found that when ball milling was performed at processing temperatures below 250 °C, the material’s electromagnetic performance was primarily influenced by plastic deformation. However, when the temperature during ball milling exceeded 250 °C, the electromagnetic characteristics were then governed primarily by grain growth. The most favorable results were obtained when the powder was subjected to heat treatment for 15 h at 250 °C. When integrated into a 1.4 mm thick wax ring sample at an 18 vol.% volume fraction in the central portion, the lamellar carbonyl iron produced in this manner demonstrated a reflectance loss of less than 8 dB across the 8–18 GHz frequency range, illustrating its exceptional broadband absorption capabilities [18]. In a similar investigation, We and their research team explored the effects of pre-heating procedures on lamellar carbonyl iron powders prior to high-energy ball milling processes. They examined the effects of various pre-heating temperatures (150 °C, 200 °C, 250 °C) and process durations (2 h, 3 h, 4 h) on the microwave absorption performances of the resulting lamellar carbonyl iron powders. Their results indicated that the most favorable microwave absorption performance was realized when the pre-heating was performed at 200 °C for 2 h [86]. These research results collectively underscore the positive effects of heat treatments in the temperature range from 200 °C to 300 °C in enhancing the magnetic permeability of lamellar carbonyl iron and in broadening its absorption bandwidth.

**Figure 6:**
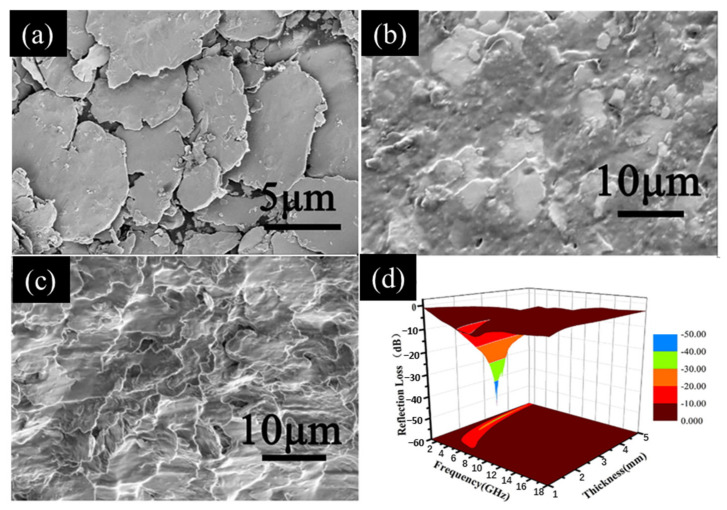
(**a**) SEM image of carbonyl iron powder after ball milling. (**b**) SEM image of a non-oriented carbonyl iron powder cross section. (**c**) SEM image of an oriented carbonyl iron powder cross section. (**d**) Relationship between the *RL* and frequency for the composite material with the 60 min orientation time [14].

Furthermore, the macroscopic alignment of these lamellar carbonyl iron powders has a significant effect on their microwave absorption capabilities. Guo and colleagues studied the way in which the orientation of lamellar carbonyl iron under application of an external magnetic field affected its absorption performance. Initially, they prepared their lamellar carbonyl iron samples using a straightforward planetary ball milling process. Subsequently, they subjected the lamellar carbonyl iron, which was embedded within a paraffin-based matrix, to an externally applied magnetic field for orientation purposes (as illustrated in Figure 6a–c). The results of their study indicated that as the orientation time increased, the real and imaginary components of the dielectric constant both decreased, while the real and imaginary parts of the magnetic permeability increased simultaneously. At the 60 min orientation mark, the complex dielectric constant reached a minimum, and the complex magnetic permeability reached a maximum. Using a specimen with a thickness of 3.25 mm, they achieved an *RL* value of −53.10 dB at 2.09 GHz, as depicted in Figure 6d. The material’s effective absorption bandwidth spanned from 1.54 to 2.93 GHz in this case, showing significant absorption efficiency within the lower frequency range [14].

Yang and colleagues prepared a flaky carbonyl iron powder and added it to epoxy resin to create several composite samples with different absorber contents. Their investigation showed that increasing either the absorber content or the aspect ratio of the powder caused the *RL* to shift towards lower frequencies [51]. However, Zhang and co-workers found that when the absorber content in the matrix approaches a saturated state (from 50 vol.% to 55 vol.%), the composite material then exhibits behavior similar to that of metals. This occurs because the eddy current losses increase, causing the magnetic permeability to decrease. Simultaneously, the dielectric constant increases significantly, which causes impedance mismatch and leads to the reflection of most of the incident electromagnetic waves [87]. In their quest to improve the impedance matching performance effectively, He and his team experimented with various mixtures of spherical and flake-shaped carbonyl iron powders at different mass ratios (*M*_flake_:*M*_sphere_ = 1:0, 2:1, 1:1, 1:2, and 0:1). They then integrated these blends into coaxial wax rings while maintaining a 30% volume fraction and assessed both their electromagnetic properties and their microwave absorption capabilities. The findings of the study indicated that although the introduction of the spherical powder shifted the resonance frequency to higher ranges, it also caused a widening of the absorption bandwidth. Notably, their composite achieved its highest *RL* when the mass ratio used was 1:1. This demonstrates that the incorporation of spherical powders can mitigate the space charge polarization effects of flake-shaped powders, fine-tune the electromagnetic properties, and optimize the impedance matching properties of the composite [88].

#### 3.1.3. Fiber/Branch-Like Absorbers

**Figure 7:**
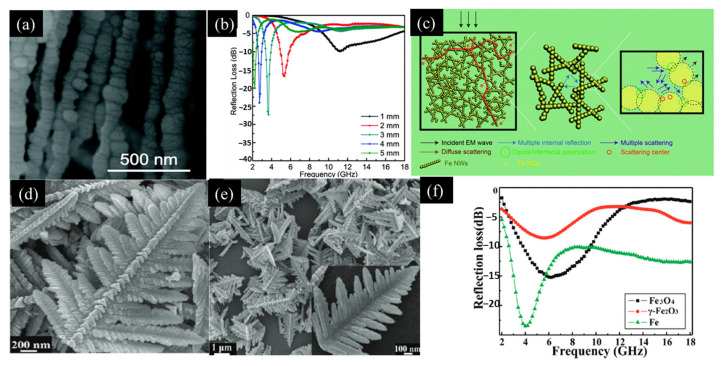
(**a**) SEM image of Fe nanowires. (**b**) Changes in the reflectance loss characteristics of Fe nanowire/paraffin composite materials with frequency. (**c**) Schematic diagram of the multi-stage enhanced microwave absorption mechanism of the Fe nanowire [90]. (**d**) SEM image of Fe_3_O_4_ dendritic microstructures. (**e**) SEM image of γ-Fe_2_O_3_ dendritic microstructures. (**f**) Imaginary parts of the complex permeability for the Fe_3_O_4_, γ-Fe_2_O_3_, and Fe dendritic microstructures [92].

One-dimensional linear iron structures, e.g., iron nanofibers, nanowires, and nanochains, offer advantages in terms of their small sizes and large surface areas. These characteristics can enhance the material’s resonant frequency and its anisotropy [10]. Researchers led by Li and their team used a chemical reduction method and controlled the reaction temperature to synthesize these linear iron nanoparticles. Their results indicated that, when compared with iron nanospheres, their iron nanowires showed significant improvements in both their magnetic permeability and their dielectric constant. The best *RL* achieved in their work was −32 dB at a frequency of 1.3 GHz with a matching thickness of 3.5 mm. The iron nanowires realized in this case demonstrated excellent absorption capabilities, particularly in the L-band range from 0.8 to 2.11 GHz [89]. Shen and colleagues used a magnetic field-assisted hydrothermal technique to synthesize high-aspect-ratio chain-like Fe nanowires with an average diameter of 100 nm, as shown in Figure 7a. The introduction of an external magnetic field was found to be essential to enable the successful growth of these linear structures. Their research also explored the effects of the precursor concentration, reaction time, and process temperature on the structural integrity of the resulting nanomaterials. The most favorable Fe nanowire morphology was achieved under the conditions of 30 mM molar concentration, 30 min reaction time, and a reaction temperature of 353 K. With regard to their microwave absorption capabilities, these Fe nanowires demonstrated higher magnetic permeability and a higher dielectric constant when compared with regular Fe particles. At a coating thickness of 3 mm, the peak *RL* reached −27.28 dB at 3.68 GHz, as illustrated in Figure 7b. The exceptional microwave absorption capabilities of these Fe nanowires were attributed to several key factors: (1) three-dimensional conductive network (the mutual interconnections of these nanowires form a three-dimensional conductive network that amplifies the dielectric loss significantly); (2) internal reflection and scattering (these nanowires are essentially composed of numerous small microspheres that are linked together, with multiple instances of internal reflection and scattering phenomena occurring within the material’s substructures as a result); and (3) contribution of rough surfaces (the irregular surface textures of the Fe nanowires provide an additional contribution to the microwave absorption performance because they cause diffuse reflection, as depicted in Figure 7c) [90].

In addition to the one-dimensional nanowire structures, branched structures have also been researched extensively. These branched structures have hyperbranched architectures with multiscale nanoscale features, endowing them with a superior electromagnetic performance when compared with other nanomaterials. Yu and colleagues synthesized large-scale three-dimensionally branched α-Fe structures, with each structure having two different surfaces with uniform widths of approximately 3.0 μm and lengths of approximately 9.0 μm. These structures exhibited enhanced dielectric constants and magnetic permeability because of a combination of orientation polarization and polycrystalline structure effects. When 70 wt.% of the absorber material was dispersed in paraffin to fabricate composite materials with a thickness of 1.9 mm, the minimum *RL* of these materials reached −32.3 dB at 10 GHz. Moreover, the branched Fe structures exhibited significantly wider absorption bands than conventional materials, especially within the mid-frequency range of 6–14 GHz, where their maximum absorption bandwidths extended to nearly 12 GHz at levels below −10 dB [91]. Sun and their team pioneered a technique for the synthesis of branched α-Fe_2_O_3_ structures using a hydrothermal method. Subsequently, they fabricated branched Fe particles, branched Fe_3_O_4_ particles, and branched γ-Fe_2_O_3_ particles through a high-temperature hydrogen reduction procedure, as depicted in Figure 7d–f. These three materials collectively demonstrated exceptional microwave absorption capabilities, particularly in the low- and mid-frequency ranges (2–9 GHz) [92].

In summary, porous microspheres and flaky iron particles have attracted significant attention due to their excellent microwave absorption properties. By adjusting the parameters during the preparation process, such as Fe^3+^ concentration and corrosion time, the morphology of porous microspheres can be precisely controlled. While the flaky iron particles are typically prepared by ball milling technology, optimizing the preparation process to adjust the particle shape, grain size, dielectric constant, and permeability, thereby enhancing absorption performance within specific frequency ranges. The preparation of composites further expands their applications by optimizing particle arrangement and content to improve absorption efficiency and bandwidth. Additionally, researchers prepared nanometer absorbing materials through situ reduction, hydrothermal method, pyrolysis, etc. Novel nanomaterials demonstrate potential advantages through multi-scale features and magnetic field control, enhancing microwave absorption capabilities. The future research direction should focus on a refined material structure design and multi-scale regulation to promote the further performance optimization and wide application of microwave-absorbing materials, meeting the demands of various frequency bands and application scenarios.

### 3.2. Multi-Interface Coupling and Heterostructure Design and Absorption Performance

In addition, other researchers have created composite materials by combining iron particles with various substances and designing a diverse range of heterostructures because of the limited absorption mechanisms and the suboptimal impedance matching performance provided by pure iron-based absorbents. These strategies enhance the absorption efficiency effectively, broaden the range of frequencies over which absorption occurs, reduce the absorber’s weight, and allow the objectives of having an absorber with a thin profile, wide absorption bandwidth, low mass, and high structural strength to be achieved. The following sections outline several commonly used synthesis methods for these composite materials.

#### 3.2.1. Doping Method

**Figure 8:**
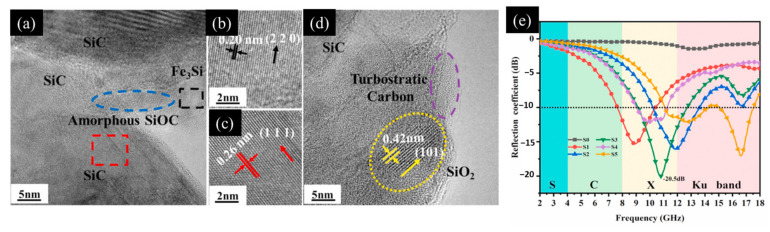
(**a**) Transmission electron microscope (TEM) image of Fe-doped SiOC ceramics. (**b**) TEM image of Fe_3_Si. (**c**) TEM image of β-SiC. (**d**) TEM image of SiO_2_ and disordered carbon. (**e**) Variation of the reflection coefficient (RC) with frequency for samples with different Fe contents at a thickness of 2.8 mm [93].

It has been verified that the direct introduction of iron into the relevant compound can increase the various loss mechanisms effectively and also enhance the material’s electromagnetic wave absorption capability. Ma and colleagues used [7] a polymer-derived ceramic method to synthesize Fe-doped SiOC ceramics at a high temperature of 1500 °C and subsequently investigated the electromagnetic properties of these ceramics within the 2–18 GHz frequency range. Their results showed that the accurate control of the iron content enables the adjustment of the compositions and quantities of multiphase products, including Fe_3_Si (the black dotted area), SiC (the red dotted area), SiOC (the blue dotted area), and disordered carbon, as illustrated in Figure 8a. Fe plays a crucial role in inducing grain size variations, while Fe_3_Si enhances the magnetic losses significantly (Figure 8b). The formation of SiC (Figure 8c) and disordered carbon during the fabrication process also increases the polarization and electrical conduction losses significantly. Furthermore, the presence of the Fe_3_Si magnetic particles and the SiO_2_ (Figure 8d) dielectric particles improves impedance matching, thereby elevating the material’s electromagnetic wave absorption performance. At an Fe content of 3 wt.% and a thickness of 2.8 mm, this composite material achieved a remarkable minimum *RL* of −20.5 dB at 10.8 GHz, as depicted in Figure 8e, thus demonstrating its outstanding absorptive characteristics within the X-band frequency range [93]. Wang and his team conducted a hydrothermal synthesis of cerium hydroxycarbonate (CeOHCO_3_), which was doped with Fe/Co/Ni. The structure, defects, shape anisotropy, impedance matching characteristics, and electromagnetic wave absorption capacity of the resulting composites were controlled by varying the doping levels of Fe/Co/Ni. When compared with pure CeOHCO_3_, CeOHCO_3_ doped with 10% Fe exhibited an excellent low-frequency absorption performance, realizing an *RL* of −47.22 dB at 2.4 GHz in combination with a broad absorption bandwidth of 3.47 GHz. This performance improvement can be attributed to the introduction of Fe, which leads to the formation of laminated dendrites. By varying the doping content, the internal stress and the oxygen vacancy defect content can be adjusted, thus improving the material’s impedance matching and attenuation capabilities [94].

Furthermore, mixing iron magnetic powder with other absorbing materials or mixing two different types of iron magnetic powder represents the simplest ways to manufacture iron-based composite absorbents. Zheng et al. used a high-energy ball milling method to prepare a lamellar carbonyl iron powder (CIP) and subsequently applied ultrasonic mixing to create CIP/FeSiAl composite materials with various mass ratios. By adjusting the mass ratios of CIP/FeSiAl, it becomes feasible to adjust the absorption properties of these composites for operation in the S-band. Their results demonstrated that, at a mass ratio of 1:4, the *RL* reached a minimum value of −6.4 dB at 2.3 GHz [95]. Tian et al. used a wet ball milling method to blend graphene, TiO_2_, and ZnO with lamellar FeSi. Their results indicated that the morphologies of graphene, TiO_2_, and ZnO had a more significant impact on the electromagnetic performance of the composite material than the intrinsic characteristics of these materials. The best microwave absorption performance was reached with the addition of TiO_2_. Specifically, when the absorber thickness was 1.5 mm, a minimum *RL* of −14.1 dB was achieved at 7.1 GHz with an associated absorption bandwidth of 3.6 GHz (*RL* < −10 dB). Additionally, when the absorber thickness was increased to 2.4 mm, the minimum *RL* reached −35.3 dB at 4.2 GHz [96].

#### 3.2.2. Coating Method

Coating represents an effective technique for the surface modification of materials. Researchers commonly enhance the performances of Fe-based absorbers by depositing various materials onto their surfaces to establish an efficient conductive network, and improve their impedance matching characteristics.

##### Carbon Coating

**Figure 9:**
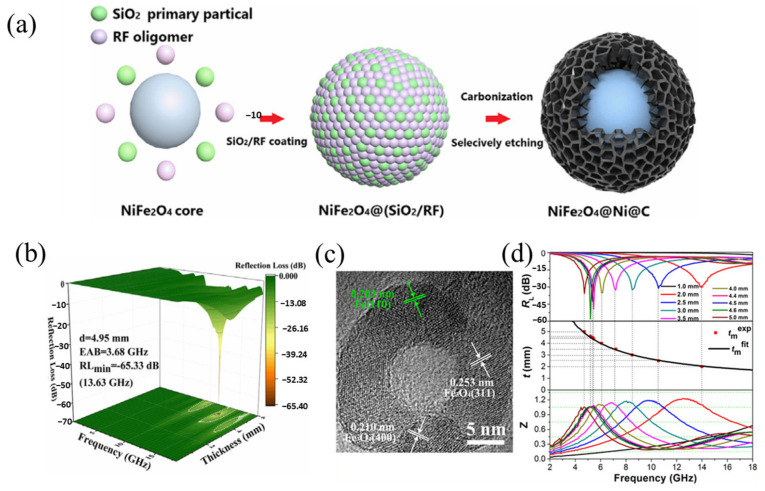
(**a**) NiFe_2_O_4_@Ni@C preparation process diagram. (**b**) Variation of the *RL* characteristics of a wax composite coating with different thicknesses with frequency when the ethanol/water volume ratio is 5/1 [97]. (**c**) High-resolution TEM (HRTEM) image of hollow Fe_3_O_4_-Fe/G composites. (**d**) Absorption performance diagram of Fe_3_O_4_-Fe/G for *d* = 1–5 mm [98].

Because of the inherent oxidation sensitivity, the singular electromagnetic loss behavior, and the suboptimal impedance matching performance of pure iron particles, researchers usually use a strategy that involves encapsulating these particles with carbon materials to design core–shell structures and thus greatly improve their electromagnetic properties. Liu et al. synthesized a nickel ferrite composite material, denoted by NiFe_2_O_4_@Ni@C, which featured a honeycomb-like carbon structure that encapsulated the outer layer, via a combination of hydrothermal treatment, in situ polymerization, and calcination processes (Figure 9a). Their findings indicated that the composite material reached a minimum *RL* of −65.33 dB at a thickness of 4.95 mm, which corresponds to an effective absorption bandwidth of 3.68 GHz (Figure 9b). The composite’s exceptional microwave absorption performance can be attributed to multiple factors: the multiple reflections and scattering induced by the material’s honeycomb-like pores and its core–shell structure, interfacial polarization arising from the heterogeneity of the materials, magnetic losses generated by the nickel core, and dipole polarization that stems from defect functional groups and oxygen vacancies [97].

Lei et al. developed a novel approach for the synthesis of lily-like hollow graphene microspheres (HGSs) by using an oil-in-water microemulsion (ME) method. Subsequently, the HGSs/Fe_3_O_4_ composites were fabricated via an in situ thermal reduction process. The unique hollow and porous structures of the HGSs provided an increased specific surface area, which is advantageous for enhanced impedance matching. Moreover, the presence of wrinkled graphene sheets allowed extensive conductive networks to be established, thereby augmenting the electrical conductivity loss. Additionally, the formation of heterojunctions between the graphene sheets and the Fe_3_O_4_ nanoparticles induced polarization relaxation, which substantially contributed to the dielectric loss and thus improved the material’s overall electromagnetic absorption properties. As a result of the synergistic effects of the mechanisms described above, the composite material exhibited superior low-frequency performance when compared with conventional materials. At a thickness of 5 mm, the material achieved an *RL* of −24.87 dB at 5.08 GHz. By adjusting the sample thickness over the range of 1.5 mm to 5 mm, the absorption bandwidth of the HGS/Fe_3_O_4_ composite can be extended to cover a wide range of 13.5 GHz (4.5–18 GHz), thus demonstrating its potential for use as an effective broadband electromagnetic wave absorber [99]. The hollow structures discussed in these works are predominantly in the micrometer range. Prior studies have demonstrated marked enhancements in electromagnetic wave absorption properties when the grain sizes of the absorptive materials are scaled down toward the nanometer scale [100,101]. Qu and colleagues engineered a nanocomposite material by integrating Fe_3_O_4_-Fe nanoparticles with graphene sheets; this material was referred to as Fe_3_O_4_-Fe/G (Figure 9c). The nanosized spheres present within this composite are capable of inducing greater numbers of dipoles both inside the hollow nanoparticles and on the particle surfaces, which leads to amplified dipolar polarization. Their composite was replete with numerous heterogeneous interfaces that enhanced the electromagnetic wave absorption capabilities of the material through increased interfacial polarization and relaxation phenomena. When incorporated at 18 wt.% into a paraffin matrix and at a material thickness of 2 mm, the composite realizes an optimal absorption bandwidth of 6.2 GHz, with its *RL* reaching −30.5 dB. At a thickness of 4.6 mm, the composite exhibited an exceptional peak *RL* of −58.0 dB (Figure 9d) [98].

##### Metal Coating

Researchers frequently use metal cladding approaches to augment the dielectric loss properties of iron-based wave absorbers while also bolstering their resistance to both oxidation and corrosion. Yang and his team successfully coated Fe nanowires with Ag using a liquid-phase reduction technique. The presence of the Ag nanoparticles serves to intensify both the interface polarization and the dipole polarization, thus leading to an enhanced dielectric loss. When adjusting the molar ratio of Fe to Ag to fine-tune the Ag shell thickness, it was observed that the electromagnetic performance of the composite material was optimized most notably when the ratio of Fe to Ag was set at 2:1. When it was incorporated into a wax matrix at a concentration of 25 wt.%, the Fe@Ag composite achieved its peak performance. At a matching thickness of 3.36 mm, the composite attains a minimum *RL* of −58.69 dB at a frequency of 7.53 GHz. Furthermore, this composite material boasts an effective absorption bandwidth (EAB) that spans an impressive 7.32 GHz [102]. Li et al. used a chemical plating method to coat spherical CIP with Ni and Co. Their results revealed that the CIP@Co composite had a relatively minor impact on the electromagnetic loss. At a thickness of 1.65 mm, the composite achieved a minimum *RL* of −57.93 dB at 12.4 GHz with an EAB of 8.56 GHz. After continuous immersion in seawater for 960 h, this composite, with a reduced thickness of 1.56 mm, exhibited an *RL* of −71.11 dB. These results clearly demonstrate that the corrosion resistance of the CIP@Co composite is significantly superior to that of most other corrosion-resistant wave-absorbing materials [103].

##### Conductive Polymer Coating

**Figure 10:**
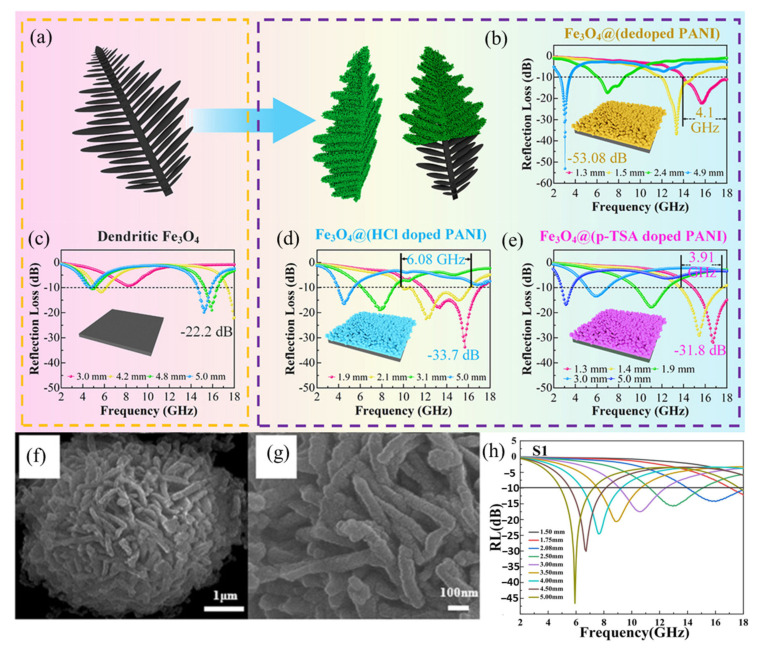
(**a**) Diagram of the coating process. (**b**) *RL*–frequency (*f*) curve of Fe_3_O_4_@(dedoped PANI). (**c**) *RL*–*f* curve of Fe_3_O_4_. (**d**) *RL*–*f* curve of Fe_3_O_4_@(HCl dedoped PANI). (**e**) *RL*–*f* curve of Fe_3_O_4_@(p-TSA dedoped PANI) [104]. (**f**,**g**) SEM images of Fe_3_O_4_@PPy. (**h**) *RL*–*f* curve of Fe_3_O_4_@PPy [105].

The integration of iron-based wave-absorbing materials with conductive polymers can simultaneously achieve both weight reduction and an improvement in the material’s impedance matching performance. Luo et al. synthesized dendrite-like Fe_3_O_4_/polyaniline (PANI) composites using a combination of hydrothermal and thermal reduction methods. As a result of the combination of induced interfacial and dipole polarizations with the establishment of a conductive network, this composite material demonstrated superior wave absorption capabilities. With a sample thickness of 1.3 mm, the material achieved an RL of −53.1 dB at a frequency of 3.0 GHz, and it also presented an EAB of 4.1 GHz (Figure 10a–e) [104]. Tian et al. constructed three-dimensional flower-like Fe_3_O_4_@PPy core–shell composites using a similar fabrication approach. The incorporation of a magneto-electric cooperative effect endowed their material with exemplary microwave absorption characteristics. The core–shell composite achieved a peak *RL* of −46.6 dB at 5.92 GHz, indicating its remarkable microwave absorption capabilities at a filler load of 30% (Figure 10f–h) [105].

##### Oxide Coating

Currently, the two most prevalent oxide coating types are semiconductor material coatings and metal oxide coatings. Semiconductor material coatings, which include SnO_2_, SiO_2_, ZnO, and MnO_2_, are known to offer chemical stability and excellent dielectric properties. The application of a semiconductor material coating to the exterior of iron-based wave absorbers can lower the material’s dielectric constant effectively, which in turn enables the fine-tuning of the impedance matching for enhanced performance. Kuchi et al. synthesized a nanocomposite material, denoted by Fe_3_O_4_@SnO_2_, with an urchin-like core–shell structure using a hydrothermal deposition process. Their results indicated that this material could achieve an *RL* of −66.5 dB at 6.8 GHz (with 30 wt.% loading and a thickness of 3 mm), and it also exhibited an EAB of 5.6 GHz [106]. Yang and colleagues used the ball milling method to fabricate flake-like FeSiCr powders that were subsequently encapsulated using a SiO_2_ layer. This process not only bolstered the electromagnetic attributes of the material but also enhanced its oxidation resistance significantly. The SiO_2_ encapsulation served to insulate the conductive network on the flake-like FeSiCr surfaces, thus reducing the overall material conductivity and effectively mitigating any eddy current effects. Their findings showed that the material could reach a notable *RL* of −65.3 dB at 4.4 GHz with an EAB of 1.9 GHz, thus demonstrating an impressive microwave absorption performance within the lower frequency spectrum [107]. Yeongjun et al. fabricated porous, flower-shaped FeCo-MnO_2_ core–shell nanoparticles and demonstrated that the material’s complex dielectric constant rose incrementally in tandem with the MnO_2_ shell’s thickness. At a frequency of 17 GHz and with a sample thickness of 1.9 mm, this material demonstrated a significant *RL* of −36 dB. This performance was ascribed to the intricate interplay of the multiple scattering and reflection phenomena produced by the combination of the composite’s porous architecture and its floral morphology, alongside the interfacial polarization and the synergistic magneto-dielectric coupling that occurred within the material [108].

The ferrite coating deftly balances high electrical resistivity with an ability to preserve the material’s magnetic characteristics, thereby increasing the electromagnetic wave absorption efficacy of the composite material. Min et al., through the application of a surface oxidation method, successfully developed flake-like carbonyl iron/Fe_3_O_4_ (FCI/Fe_3_O_4_) composites. Their research demonstrated that these FCI/Fe_3_O_4_ composites achieved both heightened absorption efficiency and an extended bandwidth. At a material thickness of 1.5 mm, the composite demonstrated an effective wave absorption bandwidth of 13.3 GHz, over the range of 4.7 to 18 GHz, with an *RL* that was maintained to be below −5 dB [109]. In addition, Al_2_O_3_, which is recognized for its low electrical conductivity, is frequently used to encapsulate soft magnetic materials. Zhou and their team synthesized FeSiAl/Al_2_O_3_ composites via a hot-press sintering process and studied the correlation between the particle size of the powder and its wave absorption capabilities. Upon adjusting the FeSiAl particle size to be within the 25–48 μm range, the composite exhibited an optimal *RL* of −34.4 dB at a frequency of 11.7 GHz. The modulation of the composite’s thickness enabled the fine-tuning of its EAB, thus demonstrating this composite’s superior tunability characteristics [110].

In investigating the electromagnetic wave absorption properties of ferromagnetic composites, researchers have employed various innovative strategies to overcome the limitations of conventional pure iron absorbers. By introducing heterogeneous structure designs, such as doping methods, metal coatings, and conductive polymer coatings, the electromagnetic properties of iron-based materials have been successfully optimized. These techniques not only extend the absorption frequency range and improve absorption efficiency but also reduce material weight and enhance structural strength. In particular, the application of metal coatings and conductive polymers has effectively enhanced the impedance matching characteristics of the materials, resulting in excellent absorption performance across a wide frequency range. Additionally, IBMA is prone to corrosion and degradation under extreme conditions, such as high temperatures and humidity, which impairs its wave-absorbing capabilities. To improve its stability for practical applications, researchers have conducted thorough investigations. Presently, techniques like chemical vapor deposition [111], containing a sol–gel method [112], grafting, and interfacial polymerization [113] are employed to encapsulate IBMA, thus enhancing its resistance to corrosion and stability at high temperatures. In summary, ferromagnetic composites are capable of achieving excellent microwave absorption properties through the application of various synthesis methods and coating technologies during the design and synthesis process, which provides a broad prospect for their application in military, communication, and electronic devices, respectively.

## 4. Magnetic Elastomer Composites

Typically, microwave-absorbing coatings and some rigid absorbing materials fall short in terms of their ability to meet mechanical strength and extensibility requirements. To solve these limitations within the existing absorber research, one popular strategy is to combine electromagnetic wave-absorbing powders with compatible elastomers to create composite materials [114,115]. Examples of such elastomers include silicone rubber, ethylene propylene diene monomer (EPDM) rubber, and various gels. These material combinations help to alleviate the mechanical limitations of the absorbers by using the inherent flexibility and resilience of the elastomer matrix. Moreover, due to its highly tunable structure, researchers can fabricate it into films or porous shapes, significantly contributing to the lightweight design of absorptive devices.

### 4.1. Rubber-Based Composites

Rubber-based microwave-absorbing materials not only offer excellent microwave absorption capabilities but also combine flexibility characteristics with a high shape recovery rate. At present, these materials are typically fabricated in sheets with specified thicknesses. Rezazadeh and his team constructed a microwave-absorbing sheet by embedding a polypyrrole/silicone rubber (PP-SR) matrix with a mixture of nano-carbon and CIP. Their research included an in-depth study of the mechanical robustness and the microwave absorption efficiency of this composite material. Their results indicated that the composite’s absorption properties peaked when the 1 mm thick PP-SR substrate incorporated a mixture of 3 wt.% nano-carbon and 47 wt.% CIP, and it achieved an *RL* of −13 dB at 10.27 GHz. In addition, the material’s *RL* remained below −7.5 dB across the entire X-band, and the material also demonstrated a commendable average tensile strength of approximately 2.96 MPa [115].

The microwave absorption efficiency of rubber-based absorption sheets is mainly dependent on the volume or weight ratio of the filler [29], and it depends on the thickness of the absorption sheet [24] when the filler content remains constant. Feng et al. synthesized a rubber-based radar-absorbing material (RAM) by integrating CIP with EPDM to act as the absorptive medium and the matrix, respectively, through a precise compounding process. They conducted a comprehensive investigation of the effects of the carbonyl iron’s volumetric fraction and the thickness of the RAM on the absorption performance within a broad frequency spectrum ranging from 2.6 to 18 GHz. Their results showed that, at a fixed thickness, an increase in the volumetric fraction of CIP produced a corresponding enhancement in the *RL* of the absorptive material, with the absorption peak shifting concomitantly towards the lower frequency domain. Specifically, a rubber absorber sheet with a thickness of 3.0 mm exhibited an *RL* that reached −21.7 dB at a frequency of 3.5 GHz at a CIP volume fraction of 45%. However, when the volume fraction remained unchanged, the absorptive efficiency of the material did not maintain a linear relationship with its thickness [24]. However, when certain rubbers are subjected to thermal decomposition or excessive cross-linking at elevated temperatures, their matrix structures become compromised. This alteration leads to a change in the distribution of the internal microwave-absorbing fillers. Moreover, it diminishes the protective role of the rubber matrix towards the fillers, resulting in their oxidation at high temperatures and a consequent reduction in their absorptive capabilities [32]. Therefore, the development of high-temperature-resistant rubber-based wave-absorbing materials represents a significant direction for future research.

### 4.2. Gel-Based Composites

An aerogel is a material with a three-dimensional network structure that is known for its high porosity, low density, and extensive specific surface area, and it is produced from wet gels [116]. In recent years, composite aerogels have received extensive attention from researchers because of their excellent microwave absorption properties. Example materials include cellulose aerogels [117], aramid nanofiber aerogels (ANFs) [118], polyimide aerogels [119], graphene aerogels [120], and MXene aerogels [121]. Among these materials, it is highly important to study the aerogels that have been modified using magnetic powders to develop high microwave absorption materials.

**Figure 11:**
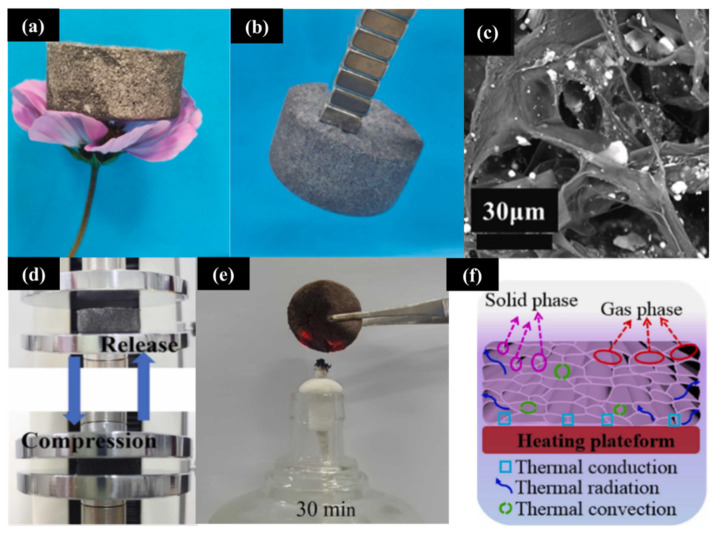
(**a**,**b**) Digital images of the TABC aerogel. (**c**) SEM image of the TABC aerogel [25]. (**d**) Digital images of the aerogel composites (in compression and recovery states) at 50% compression strain during compression testing. (**e**) Digital image of M-AMF burning on an alcohol lamp flame for 30 min. (**f**) Schematic diagram of the aerogel heat transfer mechanism [7].

Aerogels are regarded as the solids with the lightest weights. Because of its porous structure, an aerogel reduces the weight of materials required and improves microwave absorption, thus increasing the material’s operating bandwidth. Sui et al. developed a 3D CIP@polypyrrole (PPy) composite aerogel by using flaky CIP (FCIP), which enhanced the microwave absorption performance by improving the reflection and impedance matching characteristics. With a filler content of 33 wt.% and at a thickness of 2.2 mm, the absorption peaks of the composite reached −38.9 dB and −39.5 dB at 12.2 GHz and 14.2 GHz, respectively, and the bandwidth was 6.1 GHz [12]. Wang et al. fabricated an ultra-lightweight tellurium-doped black phosphorus nanoflake/aramid nanofiber/carbonyl iron nanopowder-based aerogel (TABC), as shown in Figure 11a–c. This type of aerogel has special dielectric properties that can increase the absorption bandwidth. The peak RL at 6.55 GHz was −55.12 dB and an ultra-wide effective bandwidth of 8.46 GHz was obtained at a thickness of only 1.9 mm [25].

In practical applications, factors such as the heat insulation and mechanical properties of materials must also be considered to meet the requirements for use in complex environments. Ma and his team prepared super-hydrophobic aramid nanofiber/multi-walled carbon nanotube/Fe_3_O_4_-based aerogels by freeze-drying and then treating the materials with dimethyl sulfoxide. This composite material demonstrated an excellent microwave absorption performance, with a peak *RL* of −45.83 dB at 5.46 GHz and at a thickness of 3.7 mm; this performance was attributed to the multiple reflections and scatterings of the electromagnetic waves produced by the material’s 3D structure and irregular pores. Furthermore, as depicted in Figure 11d,e, the aerogel demonstrated excellent mechanical resilience and flame resistance, maintaining its structural integrity with minimal damage after 30 min of combustion and also recovering its shape after 50% compression, with the stress reduced to 13 kPa [7]. Luo et al. created a CNT@CoFe_2_O_4_/polyimide aerogel that exhibited an EAB of 7.15 GHz and demonstrated superior thermal insulation and flame resistance characteristics [122]. The 3D network and high porosity of this aerogel obviously reduced its thermal conductivity, as illustrated in Figure 11f, thus making it a promising candidate material for a variety of applications because of its excellent insulating and fire-retardant properties.

In addition to aerogel materials, hydrogels, organogels, and ionogels, which are currently used as wave-absorbing materials, are also gradually gaining researchers’ attention [123]. Zhang et al. prepared a reduced graphene oxide (rGO)/α-Fe_2_O_3_ composite hydrogel with α-Fe_2_O_3_ particles uniformly dispersed on graphene nanosheets. When compared with a pure rGO hydrogel, the composite hydrogel demonstrated both wider and stronger wave absorption properties within the frequency range from 1 to 18 GHz [17]. Yang and his team conducted similar research and fabricated a 3D G/Fe_3_O_4_ hydrogel that achieved broad and strong absorption within the 2–20 GHz range and also exhibited high adsorption efficiency for the organic pollutant rhodamine B [28]. Cai et al. created a vertically aligned Co-MOF-74 derivative/polyacrylamide-based hydrogel. Their material not only reached a minimum *RL* of −51.6 dB and a maximum EAB of 5.73 GHz, but also showed enhanced thermal conductivity perpendicular to its surface, thus illustrating its dual potential for absorbing waves while also dissipating heat [124]. In summary, the electromagnetic properties of hydrogel composites can be adjusted by controlling their compositions, concentrations, and structures.

### 4.3. Summary

Elastomer-based microwave-absorbing materials boast a multitude of advantages due to the intrinsic properties of the elastic matrix combined with absorbing fillers. These materials exhibit high flexibility and elasticity, allowing for their application on a variety of surfaces and shapes without the risk of fracturing. Researchers can tailor the absorption frequency range and intensity by modifying the type of matrix, incorporating different absorbing fillers, or adjusting the thickness of the absorber. Broadband absorption capabilities can be achieved through a careful design of the material composition, which is essential for absorbing electromagnetic waves across multiple frequency bands. The elasticity of these materials also facilitates easy processing into various shapes via extrusion, molding, or injection techniques. Their fatigue resistance ensures durability in dynamic environments. Additionally, these materials can be multifunctional, providing extra features such as fire resistance and antistatic properties to meet the demands of specialized applications. These characteristics make elastomer-based composite-absorbing materials highly sought after in industries such as aerospace, military, and telecommunications.

## 5. Conclusions

This review encapsulates the principles of electromagnetic wave absorption by materials, discusses essential enhancement techniques for absorber performance, and examines the practical applications of absorbers. This paper summarizes strategies used to improve the electromagnetic wave absorption characteristics of ferromagnetic absorbers through microstructural (morphology, heterointerface coupling) and macrostructural enhancements, and also outlines the potential future research directions in this field.

For example, in the production of absorptive components, the development of a process that can consistently control the size, shape, distribution, and structure of these components has not been fully realized to date. A more in-depth understanding of how these micro- and macro-structures relate to the electromagnetic microwave absorption performance is also required. Therefore, we believe that there are several opportunities for further research into magnetic adsorbent materials within the following specialized areas.

First, refinement of the microstructural morphology design process to stabilize the ferromagnetic adsorbents. For example, during the synthesis of linear iron nanofibers via the chemical reduction approach, the manipulation of the raw material ratios is essential to realize controllable microstructural morphologies. Second, enhancement of the dielectric loss properties of magnetic composite absorbers via the optimization of their compositional and structural attributes, thus ensuring impedance matching and augmenting the synergistic interactions among the constituent materials. Third, enhancement of the macrostructural designs of wave-absorbing devices to ensure that they have robust absorption capabilities while also maintaining their adaptability to a broad variety of application contexts. Last, in light of the susceptibility of ferromagnetic absorbers to corrosion, it will be imperative to conduct advanced research into the high-temperature and corrosion resistance capabilities of these absorbers to ensure performance retention, with the additional objective of expanding their applicability across a range of diverse and challenging environmental conditions.
